# 2‑Thiouracil Antithyroid Drug Delivery with Functionalized BC_3_ Monolayers: A First-Principles Study

**DOI:** 10.1021/acsomega.5c08368

**Published:** 2025-11-03

**Authors:** Shengtian Hong, Xuan Luo

**Affiliations:** 528218 National Graphene Research and Development Center, Springfield, Virginia 22151, United States

## Abstract

2-Thiouracil (C_4_H_4_N_2_OS), an antithyroid drug (ATD) used in the treatment of Graves’ disease and thyroid storm, requires an effective drug delivery vehicle to successfully reach the thyroid gland. Using first-principles calculations, the adsorption of the 2-thiouracil molecule on pristine, Si-doped, and Al-doped BC_3_ monolayers was studied based on density functional theory (DFT). Our results show that chemisorption of 2-thiouracil is stronger on Si-doped BC_3_ than on Al-doped BC_3_, while pristine BC_3_ shows weak physisorption. On the doped monolayers, the O atom on 2-thiouracil displayed bond formation, hybridization, and stronger adsorption to the dopants compared to the S atom. We concluded that, of the 10 configurations computed, 2T-O_h_/Si-BC_3_ had the largest adsorption energy, making it the most promising configuration for future antithyroid drug delivery research. Our findings highlight how doped BC_3_ monolayers can act as effective drug delivery platforms for the antithyroid drug, potentially improving the treatment of Graves’ disease with future experimental validation.

## Graphical abstract

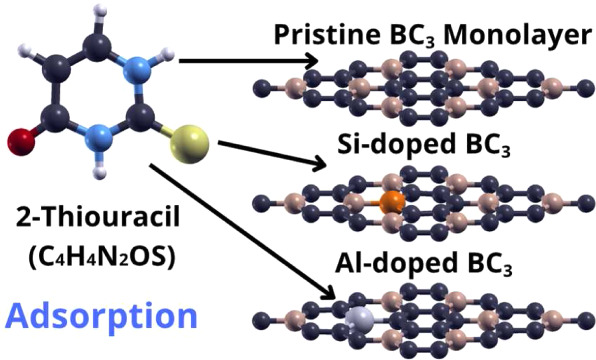

## 1 Introduction

Graves’ disease, an autoimmune condition characterized by an enlarged and overactive thyroid gland, affects millions of people globally.^1^ Although Graves’ disease itself is not life-threatening, it can lead to thyroid storm,^2^ which has a mortality rate of 25%,^3^ and potentially increase the incidence rate of thyroid cancer by 10.4 times in an individual.^4^ Therefore, research institutes and pharmaceutical industries have conducted extensive studies related to the treatment of Graves’ disease, including thyroidectomy surgery and the use of radioactive iodine.^5^ Although effective, these methods are costly and invasive, involving either surgical removal or destruction of thyroid tissue. Moreover, they carry a post-therapy hypothyroidism rate exceeding 80%.^6^ Due to these drawbacks, patients often opt for a more cost-effective and nonablative treatment method, the use of antithyroid drugs (ATDs),^6,7^ such as 2-thiouracil (C_4_H_4_N_2_OS), which require drug delivery vehicles to transport them into the thyroid tissue.^8^

Scientists have conducted extensive research on drug delivery vehicles, including zero-dimensional nanoparticles, one-dimensional nanotubes, nanocones, etc.^9,10^ In 2024, Kurban’s team aimed to use zero-dimensional (ZnO)_60_ nanoparticles for anticancer drug delivery, and they found stable and favorable interactions between these nanoparticles and therapeutics like chloroquine.^11^ Bechohra et al. discovered, in 2024, that carbon nanotubes and boron nitride nanotubes can enhance the curcumin anticancer drug’s therapeutic efficacy while minimizing its systemic toxicity.^12^ In 2023, Harismah’s group studied the potential combination of 2-thiouracil with Be-doped carbon nanocones, finding that the models were desirable for use in drug delivery against hypothyroidism.^13^

In recent years, 2D monolayers have been extensively studied as carriers for drugs due to their large surface areas and relatively small quantum sizes, which enhance their efficacy compared to other nanocarriers.^14,15^ For example, Pari and his team analyzed, in 2021, the combination of an iron-assisted carbon monolayer for the delivery of 2-thiouracil and discovered the dominant role of the monolayer in managing interactions with the drug.^16^ In 2025, Maurya et al. found the potential of using three graphyne monolayers as nanocarriers for the anticancer drug 5-fluorouracil, minimizing the drug’s toxicological damage.^17^ Within 2D monolayers, graphene is prominently used in the field of drug delivery due to its chemical stability and large surface area, which allow efficient drug loading.^18,19^ For instance, Dastani’s team concluded, in 2020, that graphene and functionalized graphene nanosheets can be used as carriers for the Cladribine anticancer drug molecule.^20^ Moreover, metal-doped graphene, as Khodadadi et al. discovered in 2019, exhibits stronger adsorption behavior to the anticancer drug beta-lapachone compared to pristine graphene, and doped graphene nanosheets can have a better effect in the delivery of the drug.^21^ Similarly, graphene-like monolayers, such as BCN,^22^ MgO,^23^ BaO,^23^ BeS,^24^ and BC_3_,^25^ all exhibit similar exceptional properties, and their implications in drug delivery have been studied.

The BC_3_ monolayer crystallizes in a hexagonal lattice with the space group *P*6/*mmm* (#191). With its exceptional chemical properties,^26^ the BC_3_ monolayer was studied by Rahimi et al. and combined with the nitrosourea anticancer drug in 2020. The results showcased a large adsorption energy, demonstrating the potential application of BC_3_ in drug delivery.^27^ In 2021, Chen et al. calculated that BC_3_ had a larger adsorption energy when combined with the anticancer drug 5-fluorouracil than that of graphene-like NC3 and pristine graphene, confirming that the boron carbide monolayer could be used as a potential carrier for drug delivery applications.^28^ Recent studies by Kurban et al. have shown that atomic-scale functionalization tunes the structural, electronic, and interfacial properties.^29–32^ Moreover, doped nanomaterials have been shown to increase the adsorption of molecules. For example, the Li_3_O–B_12_N_12_ nanocage has been studied by Sheikhi et al. and is found to be a suitable chlormethine carrier.^33^ Similarly, Kaviani’s team found that Se–B_12_N_12_ nanocluster serves as a promising drug delivery system for the ciclopirox anticancer agent.^34,35^ Specifically for BC_3_, previous studies have shown effectively increased adsorption energy, signifying stronger interactions, when molecules such as formaldehyde^36^ and acetone^37^ were combined with Si-doped and Al-doped BC_3_ compared to pristine BC_3_. Therefore, we assert that the graphene-like BC_3_ monolayer showcases high strength and conductivity, while potential isovalent substitutional doping with Si and Al could benefit the adsorption of the drug, making pristine, Si-doped, and Al-doped BC_3_ optimal monolayers for this study.

Currently, the 2-thiouracil molecule’s adsorption on pristine, Si-doped, and Al-doped BC_3_ has not been studied. While this study is theoretical, understanding adsorption behavior is a crucial step in evaluating whether functionalized BC_3_ monolayers could act as viable drug delivery nanocarriers under biological conditions. Other key factors, such as biocompatibility, stability, and targeted delivery to thyroid tissue, will ultimately determine the clinical efficacy of this system, but adsorption energy trends can identify promising configurations for *in vitro* research.

The first-principles calculations based on density functional theory (DFT)^38^ are used in this study to evaluate the effectiveness of pristine, Si-doped, and Al-doped BC_3_ monolayers as drug carriers for 2-thiouracil. Calculations were conducted to find the optimal structure of each monolayer, as well as to determine adsorption energy, band structure, total and projected density of states (DOS), and charge transfer of 2-thiouracil on pristine and doped BC_3_ monolayers. The calculated results of 2-thiouracil’s adsorption on each monolayer were then compared and analyzed to identify the optimal monolayer for the delivery of 2-thiouracil. In the second section, we explain our computational methods; in the third section, we present our results and discussions on 2-thiouracil adsorption; finally, the fourth section showcases our conclusions.

## 2 Methods

### 2.1 Computational Details

First-principles calculations were performed based on density functional theory (DFT) within the generalized gradient approximation (GGA) in the Perdew–Burke–Ernzerhof (PBE) format, as implemented in the ABINIT code.^39^ The projected augmented wave (PAW) method^40^ was used to generate the pseudopotentials of elements with the ATOM PAW.^41^ The elements in this study included hydrogen, boron, carbon, nitrogen, oxygen, aluminum, silicon, and sulfur. Table 1 shows the corresponding electron configurations and radius cutoffs of all the elements studied.

**Table 1:** Electron Configuration and Radius Cutoff r_cut_ (Bohr) of Elements Studied for Generating PAW Pseudopotential

Elements	Electron configuration	r_cut_ (Bohr)
Hydrogen (H)	1s^1^	0.995
Boron (B)	[He]2s^2^2p^1^	1.701
Carbon (C)	[He]2s^2^2p^2^	1.507
Nitrogen (N)	[He]2s^2^2p^3^	1.200
Oxygen (O)	[He]2s^2^2p^4^	1.415
Aluminum (Al)	[Ne]3s^2^3p^1^	1.904
Silicon (Si)	[Ne]3s^2^3p^2^	1.909
Sulfur (S)	[Ne]3s^2^3p^4^	1.915

For total energy calculations, the self-consistent field (SCF) iteration was considered converged when the total energy difference was smaller than 1.0 × 10^–10^ hartree twice consecutively. For accurate results, the kinetic energy cutoff, Monkhorst–Pack k-point grid, and vacuum heights were considered converged after the total energy difference of data sets was less than 0.0001 hartree (about 3 meV) twice consecutively. The converged kinetic energy cutoff was 25 hartree, the converged k-point mesh was 6 × 6 × 1 for the 2 × 2 BC_3_ supercell (equivalent to 4 × 4 graphene, 32-atom supercell), and the converged vacuum height was 25 Bohrs for all molecule-monolayer complexes. The vdW-DFT-D3 method of the van der Waals dispersion correction was used in all total energy calculations. To optimize the atomic structure, BFGS structural relaxation, a molecular dynamics method, was used. Lattice vectors were not fixed during the relaxations. The relaxation of atomic structures was considered converged when the maximum absolute force on each atom was less than 2.0 × 10^–4^ hartree/Bohr (about 0.01 eV/Å). The maximum number of iterations was 1000, while pristine BC_3_ calculations required around 100 iterations, and doped BC_3_ relaxations required 200–300 iterations.

### 2.2 Atomic Structures

#### 2.2.1 2-Thiouracil Molecule

The atomic structure of the 2-thiouracil molecule (C_4_H_4_N_2_OS), an effective antithyroid drug, is shown in Figure 1a. This planar, aromatic pyrimidine ring has a carbonyl-O group, a thione group, and two N atoms in the ring.

**Figure 1:**
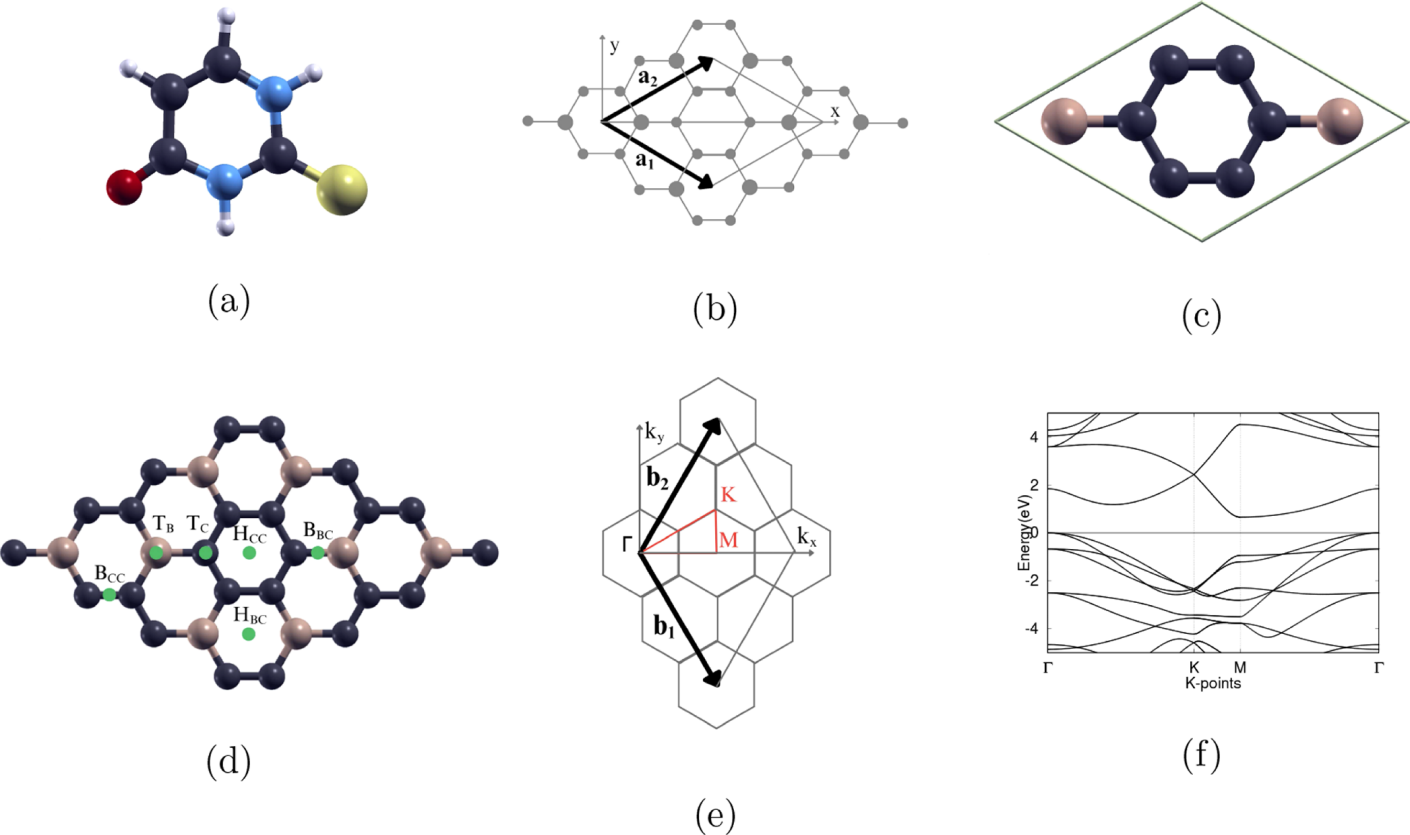
(a) The atomic structure of the 2-thiouracil (C_4_H_4_N_2_OS) molecule, (b) the Bravais lattice (**a**_**1**_, **a**_**2**_) of the BC_3_ monolayer, (c) the atomic structure of the BC_3_ primitive cell, (d) possible adsorption sites on the 2 × 2-BC_3_ monolayer, (e) the reciprocal lattice (**b**_**1**_, **b**_**2**_) of the BC_3_ monolayer, with the high-symmetry k-points in the first Brillouin zone Γ (0, 0, 0), K (1/3, 2/3, 0), and M (1/2, 1/2, 0), and (f) the band structure of the BC_3_ monolayer, with the Fermi level set to 0 eV and the high-symmetry k-point circuit in the following order: Γ → K → M → Γ. For all atomic structures, hydrogen, nitrogen, oxygen, sulfur, carbon, and boron atoms are represented as white, blue, red, yellow, purple, and beige, respectively.

#### 2.2.2 Pristine BC_3_ Monolayer

The BC_3_ monolayer has a 2D hexagonal Bravais lattice, as shown in Figure 1b. The 8-atom primitive cell, consisting of 2 B atoms and 6 C atoms, is displayed in Figure 1c. The BC_3_ monolayer was then scaled up to a 2 × 2 supercell with 32 atoms, as shown in Figure 1d. Figure 1e displays the reciprocal lattice of the BC_3_ monolayer, and the high-symmetry k-points in the first Brillouin zone are Γ (0, 0, 0), K (1/3, 2/3, 0), and M (1/2, 1/2, 0). The band structure of BC_3_, shown in Figure 1f, was computed along the k-point circuit Γ → K → M → Γ. The direct band gap is between the VBM and CBM at the Γ point.

#### 2.2.3 Doped BC_3_ Monolayers

To potentially improve 2-thiouracil adsorption, the BC_3_ monolayer was isovalently doped by substituting a carbon atom with silicon (Si) and a boron atom with aluminum (Al) in separate instances, creating the Si-doped-2 × 2-BC_3_ (Si-BC_3_) and Al-doped-2 × 2-BC_3_ (Al-BC_3_) monolayers.

The defect formation energy (*E*_f_) is defined as

1 $$E_{f}=E_{doped-ML}-E_{pure-ML}-E_{dopant}+E_{sub}$$

where *E*_doped–ML_, *E*_pure–ML_, *E*_dopant_, and *E*_sub_ are the chemical potentials of the doped monolayer, the pure monolayer, the dopant (Si or Al), and the substituted atom (C or B), respectively. Crystalline Si, crystalline Al, graphene, and orthorhombic B were used to compute the chemical potentials for Si, Al, C, and B, respectively.

#### 2.2.4 2-Thiouracil-BC_3_ Complexes

To place the 2-thiouracil molecule on a pristine BC_3_ monolayer, adsorption sites were identified. On 2-thiouracil, the O atom of the carbonyl group and the S atom of the thione group are considered reactive sites.

All possible adsorption sites for 2-thiouracil on 2 × 2-BC_3_ are shown in Figure 1d. T_B_ is directly on top of a B atom; T_C_ is directly on top of a C atom; B_BC_ means on the B–C bond; B_CC_ represents on the C–C bond; H_BC_ stands for in the B–C ring; and H_CC_ stands for in the ring with only C atoms.

Both reactive sites on 2-thiouracil were placed onto each adsorption site on 2 × 2-BC_3_ as possible configurations.

In doped monolayers, the dopant (Si or Al) was considered the main reactive site on the monolayer. Therefore, the O atom and S atom on 2-Thiouracil were both placed on the Si dopant vertically and horizontally, resulting in four different positions on Si-BC_3_. 2-Thiouracil was placed onto Al-BC_3_ in a similar manner, but the vertical placement, when S was on top of Al, resulted in an unstable configuration, so only three positions are present.

To avoid the size effect, we converged the unit cell size for the 2-thiouracil molecule, which was 20 × 20 × 20 Bohrs. Therefore, we chose a 2 × 2-BC_3_ supercell, which had a unit cell size of 20 × 20 Bohrs. The vacuum layers of 2-Thiouracil and BC_3_ monolayers were calculated independently, and the values were added as the vacuum layer of the drug-monolayer complex while ensuring an additional buffer space. The vacuum was chosen to be 25 Bohrs to account for the distance between the molecule and the monolayer.

Total energy calculations were then run for the monolayer-molecule complex, the isolated 2-thiouracil molecule, and the isolated monolayers. Adsorption energy (*E*_ad_) was defined by

2 $$E_{ad}=E_{mol+ML}-E_{ML}-E_{mol}$$

where *E*_mol+ML_, *E*_ML_, and *E*_mol_ represent the total energies of the complex, the monolayer, and the 2-thiouracil molecule, respectively. A more negative adsorption energy demonstrates a more stable configuration, as well as stronger adsorption. Chemisorption and physisorption between the molecule and the monolayer were determined based on adsorption energy, hybridization, and charge transfer.

### 2.3 Electronic Structures

#### 2.3.1 Band Structure

The band structures for all optimized monolayers and molecule-monolayer complexes were calculated along high-symmetry k-points Γ (0,0,0), K (1/3, 2/3, 0), and M (1/2, 1/2, 0), as shown in Figure 1b.

#### 2.3.2 Density of States (DOS)

Total DOS was calculated and compared with the band structure to further analyze the distributions of the density of states at different energy levels, especially around the band gap. To further analyze the contributions of 2-thiouracil adsorption on BC_3_ monolayers to the density of states, projected DOS calculations were conducted using the tetrahedron method. The atoms chosen for projection are the interacting atoms on the monolayer and the 2-thiouracil molecule.

#### 2.3.3 Charge Transfer

The complexes that demonstrated strong adsorption were selected for charge transfer calculations. Charge transfer in the molecule-monolayer complex was determined by finding the difference in charge density between the combined materials and the separate constituents, as indicated in eq 3.

3 $$\Delta \rho \left(r\right)=\rho_{mol+ML}\left(r\right)-\rho_{ML}\left(r\right)-\rho_{mol}\left(r\right)$$

where Δρ represents the charge difference. ρ_mol+ML_, ρ_ML_, and ρ_mol_ represent the charge density of the molecule-monolayer complex, the monolayer, and the 2-thiouracil molecule, respectively. We plot the isosurface to visually display charge density differences.

## 3 Results and Discussion

The optimized atomic structures, adsorption energies, band structures, density of states, and charge transfer were calculated for the 2-thiouracil molecule adsorbed on pristine, Si-doped, and Al-doped BC_3_ monolayers using first-principles calculations.

### 3.1 Molecule and Monolayer Calculations

The calculated lattice constants for the primitive BC_3_ cell, selected bond angles for the 2-thiouracil molecule, and selected bond lengths for both the 2-thiouracil molecule and the BC_3_ monolayer are shown in Table 2.

**Table 2:** Current Calculated, Other Theoretically Calculated, and Experimental Structural Parameters of the 2-Thiouracil (C_4_H_4_N_2_OS) Molecule and BC_3_ Monolayer^a^

	Parameter	Calculated	Other	Experimental	Error(%)
2-Thiouracil	d_CO_ (Å)	1.220	1.220 ^42^	1.230 ^43^	0.810
	d_CS_ (Å)	1.650	1.670 ^42^	1.680 ^43^	1.780
	∠_OCN_ (^◦^)	119.8	120.0 ^42^	119.2 ^43^	0.500
	∠_SCN_ (^◦^)	122.4	122.3 ^42^	122.2 ^43^	0.160
BC_3_	a (Å)	5.175	5.170 ^44^	5.370 ^45^	3.720
	d_BC_ (Å)	1.570	1.560 ^44^	1.560 ^45^	0.640
	d_CC_ (Å)	1.420	1.420 ^44^	1.420 ^45^	0

a Error represents the relative difference between the current calculated and experimental data . Bond length d (Å), bond angle ∠ (◦), and lattice constant a (Å) were compared .

As shown in Table 2, for the 2-thiouracil molecule, the CO and CS bond lengths were calculated to be 1.220 and 1.650 Å, while previous calculations by Rani et al. showed similar results of 1.220 and 1.670 Å.^42^ Tiekink et al. have measured values of 1.230 and 1.680 Å^43^ in experiments, making the errors 0.810% and 1.780%, respectively. Our calculated results for the OCN and SCN bond angles are 119.8^◦^ and 122.4^◦^, which are similar to previously calculated values of 120.0 and 122.3°;^42^ Tiekink et al. showed experimental results of 119.2° and 122.2°,^43^ and the error values of 0.500% and 0.160% also confirm our calculations. Therefore, data from previous work demonstrate the reliability of our molecular calculations.

The primitive cell BC_3_ calculations show a lattice constant of 5.175 Å, a BC bond length of 1.570 Å, and a CC bond length of 1.420 Å, which is consistent with previously calculated values of 5.170 Å, 1.560 Å, and 1.420 Å by Bafekry et al.;^44^ Yanagisawa et al. measured these values to be 5.370 Å, 1.560 Å, and 1.420 Å^45^ in experiments, which result in reasonable error values of 3.720%, 0.640%, and 0%, respectively, confirming our monolayer calculations.

Overall, the currently calculated parameters for the 2-thiouracil molecule and the BC_3_ monolayer demonstrate excellent agreement with previous theoretical computations, as well as experimental data, verifying our computational methods.

The optimized atomic and band structures of pristine, Si-doped, and Al-doped BC_3_ can be found in Figure 2a,b and c, respectively. After Si or Al doping, the BC_3_ monolayer does not show significant deviation from the planar structure. All three monolayers have visible direct band gaps between the VBM and CBM at the Γ point, as shown in the band structure and total DOS.

**Figure 2:**
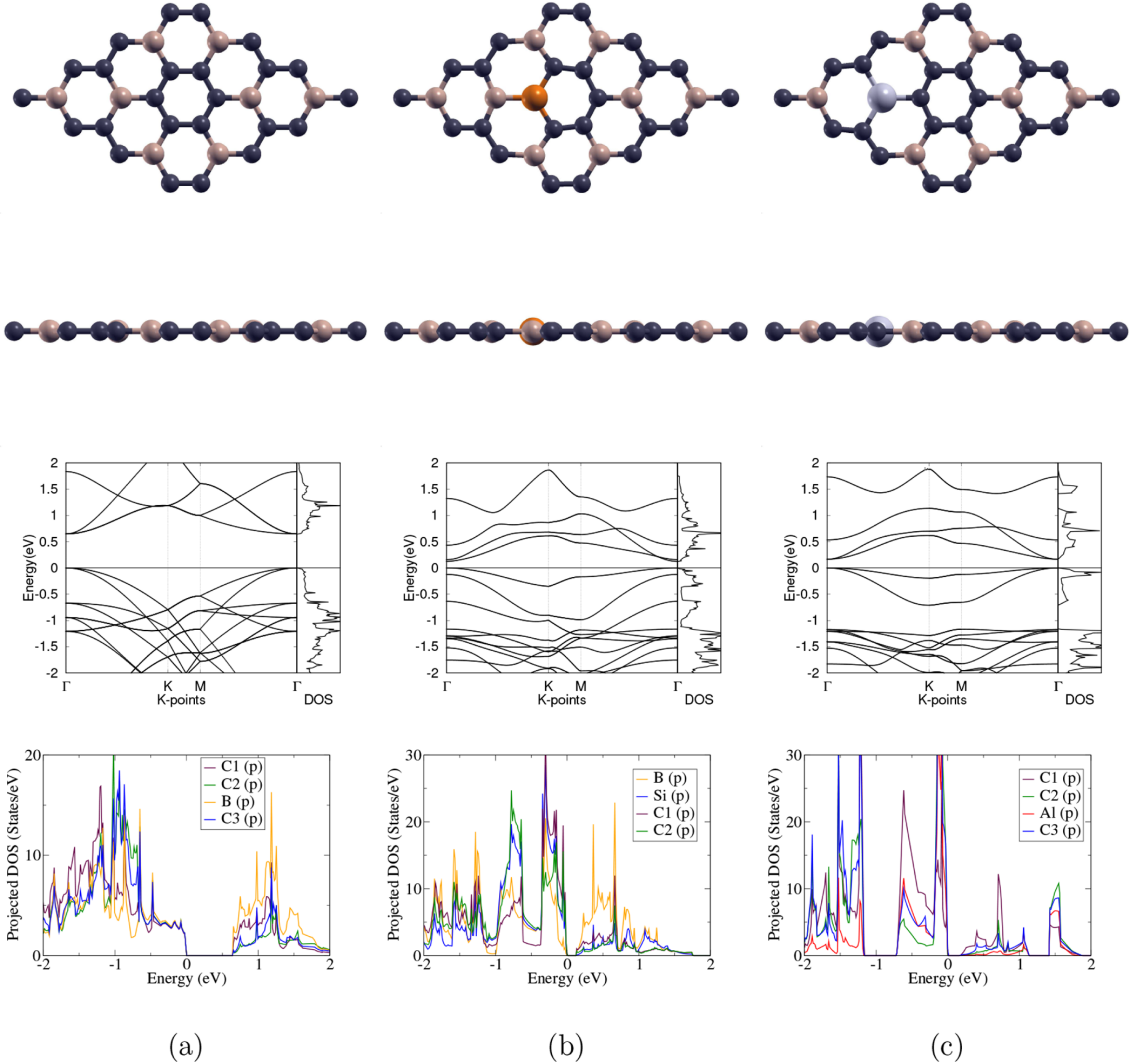
(a) 2 × 2-BC_3_, (b) Si-doped BC_3_, and (c) Al-doped BC_3_. The first and second rows show the top and side views of the optimized atomic structures of the 3 monolayers; carbon, boron, silicon, and aluminum atoms are represented as purple, beige, blue, and gray, respectively. The third row displays the band structures and total DOS of the 3 monolayers, with the Fermi level set to 0 eV; the high-symmetry k-point circuit runs in the following order: Γ → K → M → Γ. Specifically, the B_2p_ orbitals are shown for BC_3_, the Si_3p_ orbitals for Si-BC_3_, and the Al_3p_ orbitals for Al-BC_3_, along with the valence p orbitals of the three atoms directly bonded to them, respectively.

The projected DOS graphs in Figure 2 display that all orbitals contribute to the states below, above, and at the Fermi level (0 eV) for BC_3_, Si-BC_3_, and Al-BC_3_. Strong overlap in states and hybridization can be observed throughout −2 to 2 eV in the projected DOS of both pristine and doped BC_3_ monolayers.

The lattice constant, band gap, and defect formation energy of the optimized monolayers are shown in Table 3. BC_3_ has the smallest lattice constant of 10.35 Å, while Si-BC_3_ and Al-BC_3_ have larger lattice constants of 10.53 and 10.54 Å, respectively; this difference is most likely due to the larger atomic radius of Si and Al compared to a C or B atom. In contrast to the 0.648 eV band gap of pristine BC_3_, both Si-BC_3_ (0.124 eV) and Al-BC_3_ (0.166 eV) have smaller band gaps. Moreover, Si-BC_3_ has a defect formation energy of 3.311 eV, which is smaller than that of Al-BC_3_ (9.964 eV), indicating that it requires less energy to replace a C atom with a Si atom than to replace a B atom with an Al atom on the BC_3_ monolayer. The effective dopant concentration for both Si-BC_3_ and Al-BC_3_ is 3.125%, since 1 out of 32 atoms in the supercell is doped.

**Table 3:** Lattice Constant a (Å), Band Gap *E*_g_ (eV), and Defect Formation Energy *E*_f_ (eV) of Pristine and Doped BC_3_ Monolayers^a^

Monolayer	a (Å)	*E*_g_ (eV)	*E*_f_ (eV)
BC_3_	10.35	0.648 (dir)	N/A
Si-BC_3_	10.53	0.124 (dir)	3.311
Al-BC_3_	10.54	0.166 (dir)	9.964

a Dir represents the direct band gap.

### 3.2 2-Thiouracil Adsorbed on Pristine BC_3_

To place the 2-thiouracil molecule on pristine BC_3_, there are 9 potential adsorption sites, as shown in Figure 1d. In 2021, Z. Zhao et al. calculated the relaxation and adsorption of CH_3_COCH_3_ on BC_3_, and they discovered an energy-favorable position at the T_B_ site (top of a B atom) in pristine BC_3_.^46^ In a separate study by L. Zhao et al. in 2021, first-principles calculations indicated three stable adsorption sites: the hollow site of the carbon ring (H_CC_), the hollow site of the carbon–boron ring (H_BC_), and on top of a B atom (T_B_).^47^ Therefore, the T_B_, H_BC_, and H_CC_ positions on pristine BC_3_ were selected to initially place and potentially bind with reactive sites of 2-thiouracil.

Although 2-thiouracil was initially placed on T_B_, H_CC_, and H_BC_ sites, the O atom demonstrated an affinity for the T_B_ site, while the S atom showed an affinity for the H_BC_ site after relaxation. In the relaxed configuration, where 2-thiouracil was horizontal, when O was on T_B_, S was consequently pushed to a separate T_B_ site. The top and side views of the three optimized structures, 2T-O/BC_3_, 2T-S/BC_3_, and 2T-h/BC_3_, are displayed in the top two rows of Figure 3. In the 2T-O/BC_3_ configuration, the molecule is vertical, with the O atom on the T_B_ site; 2T-S/BC_3_ also represents a vertical position, with the S atom on the H_*BC*_ site; in 2T-h/BC_3_, the molecule is horizontal, and both the O and S atoms are on T_B_ sites. The computed band structures, total density of states (DOS), and projected DOS are shown below each atomic structure in Figure 3. Adsorption energy, lattice constant, band gap, and binding distance of each complex are listed in Table 4.

**Figure 3:**
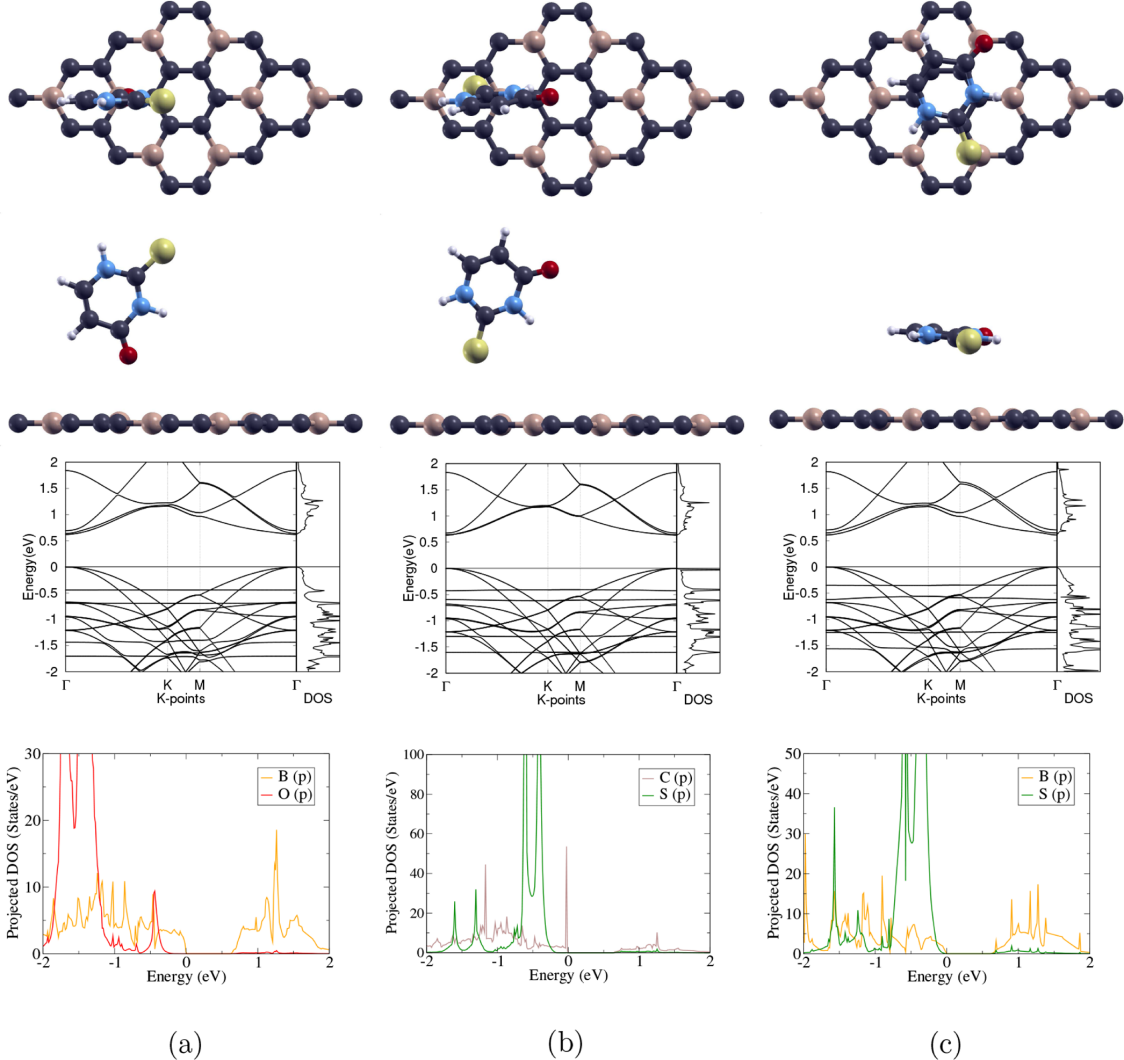
Optimized configurations of the 2-thiouracil molecule on 3 different sites of the pristine BC_3_ monolayer: (a) 2T-O/BC_3_, (b) 2T-S/BC_3_, and (c) 2T-h/BC_3_. The first and second rows show the top and side views of the 3 configurations, respectively; carbon, boron, oxygen, nitrogen, hydrogen, and sulfur atoms are represented as purple, beige, red, blue, white, and yellow, respectively. The third row displays the calculated band structures and total DOS of each configuration, with the Fermi level set to 0 eV; the high-symmetry k-point circuit runs in the following order: Γ → K → M → Γ. The last row shows the projected DOS of two selected orbitals of each configuration; the B_2p_, O_2p_, C_2p_, and S_3p_ orbitals are represented as yellow, red, brown, and green, respectively.

**Table 4:** Adsorption Energy *E*_ad_ (eV), Lattice Constant a (Å), Band Gap *E*_g_ (eV), and Binding Distance d (Å) of 2-Thiouracil on Pristine BC_3_ ^a^

Complex	*E*_ad_ (eV)	a (Å)	*E*_g_ (eV)	d (Å)
2T-O/BC_3_	–0.247 (phy)	10.35	0.619 (dir)	3.050
2T-S/BC_3_	–0.288 (phy)	10.35	0.634 (dir)	3.490
2T-h/BC_3_	–0.559 (phy)	10.35	0.611 (dir)	3.510

a Phy represents physisorption, and dir represents the direct band gap.

#### 3.2.1 2T-O/BC_3_

The optimized atomic structure of 2T-O/BC_3_ is shown in Figure 3a. The binding distance, which is the distance between the two closest atoms on the molecule and the monolayer, is 3.050 Å, as shown in Table 4.

As shown in the band structure, there is a direct band gap between the VBM and CBM at the Γ point. After 2-thiouracil adsorption, the band gap of BC_3_ decreased from 0.648 to 0.619 eV, as shown in Tables 3 and 4. This demonstrates 2-thiouracil’s influence on the electronic structure. In the total DOS, the same band gap of 0.619 eV is displayed.

As shown in the projected DOS in Figure 3a, the electron states around and above the Fermi level (0 eV) were contributed by the B_2p_ orbital. Both the B_2p_ orbital and the O_2p_ orbital contributed to the states below the Fermi level, and minimal hybridization can be found between the B_2p_ orbital and the O_2p_ orbital at around −0.5 eV. This suggests some bonding between 2-thiouracil and BC_3_, corresponding to physisorption, as indicated by the low adsorption energy of −0.247 eV shown in Table 4.

#### 3.2.2 2T-S/BC_3_

The optimized structure of 2T-S/BC_3_ is shown in Figure 3b. The binding distance is measured to be 3.490 Å, as shown in Table 4.

The direct band gap between the VBM and CBM at the Γ point, as shown in the band structure, is revealed to be 0.634 eV in Table 4. This band gap is smaller compared to the pristine BC_3_ band gap of 0.648 eV in Table 3, demonstrating changes in the electronic structure due to adsorption. The total DOS indicates the same band gap.

The projected DOS in Figure 3b shows that the electron states around and above the Fermi level (0 eV) were mainly contributed by the C_2*p*_ orbital. Moreover, both the C_2p_ orbital and the S_3p_ orbital contributed to the states below the Fermi level, and only very minimal hybridization can be found between the C_2p_ orbital and the S_3p_ orbital at around −0.7 eV, suggesting minimal interactions between the drug molecule and the monolayer, thereby confirming physisorption and the calculated −0.288 eV adsorption energy in Table 4.

#### 3.2.3 2T-h/BC_3_

The atomic structure of 2T-h/BC_3_ after relaxation is shown in Figure 3c. The binding distance in Table 4, 3.510 Å, is similar to that of the other two configurations.

A direct band gap between the VBM and CBM at the Γ point is displayed in the band structure. After 2-thiouracil adsorption on BC_3_, the band gap decreased from 0.648 to 0.611 eV, as shown in Tables 3 and 4. This minimal change demonstrates evidence of an interaction between 2-thiouracil and pristine BC_3_. The total DOS confirms the band gap of 0.611 eV.

In Figure 3c, the projected DOS shows that the electron states around and above the Fermi level, set to 0 eV, were contributed mostly by the B_2*p*_ orbital. Furthermore, both the B_2p_ orbital and the S_3p_ orbital contributed to the states below the Fermi level, and hybridization is observed at −1.6 eV, demonstrating evidence of bonding between BC_3_ and 2-thiouracil, which is also reflected by the largest adsorption energy of −0.559 eV out of all three positions shown in Table 4. However, due to the lack of strong hybridization, this adsorption is still classified as physisorption.

Overall, although 2T-h/BC_3_ has the largest adsorption energy, −0.559 eV, among the three complexes, weak physisorption is observed between the 2-thiouracil molecule and the pristine BC_3_ monolayer due to the small adsorption energies and minimal hybridization shown in the projected DOS.

### 3.3 2-Thiouracil Adsorbed on Si-Doped BC_3_

Previous studies have shown that placing molecules on the Si dopant leads to better adsorption, only the Si site was considered.^36,37^ 2-Thiouracil was placed onto Si-doped BC_3_ in four positions: 2T-O/Si-BC_3_, 2T-O_h_/Si-BC_3_, 2T-S/Si-BC_3_, and 2T-S_h_/Si-BC_3_. O and S represent the atom on 2-thiouracil placed closest to the Si dopant on the monolayer, with configurations marked “h” indicating a horizontal 2-thiouracil molecule as opposed to a vertical. Top and side views of all four optimized structures are shown in the first two rows of Figure 4. The computed band structures, total density of states (DOS), and projected DOS are displayed below each atomic structure. Adsorption energy, lattice constant, band gap, and binding distance of each complex are listed in Table 5.

**Figure 4:**
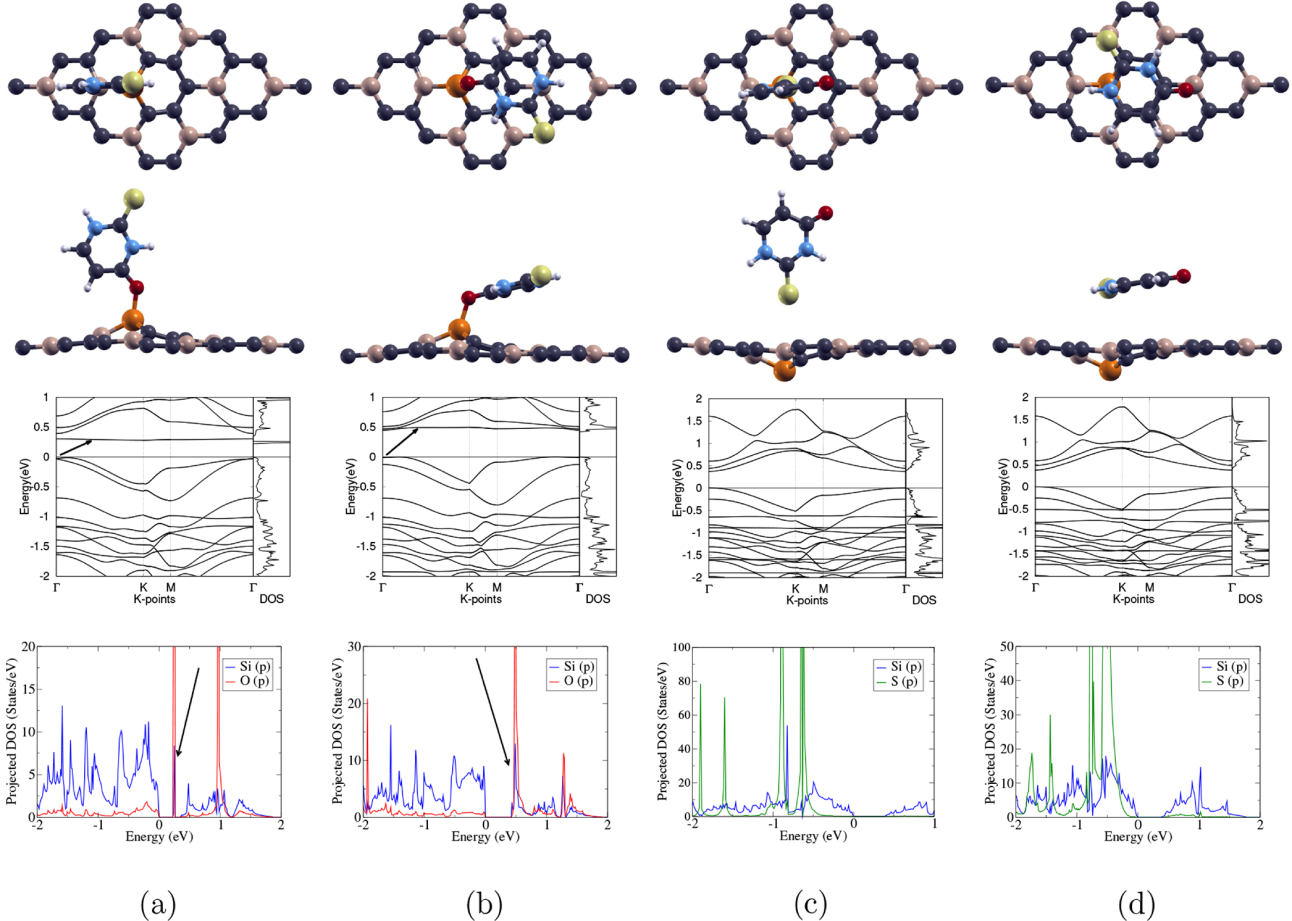
Optimized configurations of the 2-thiouracil molecule in 4 different positions on the Si-doped BC_3_ monolayer: (a) 2T-O/Si-BC_3_, (b) 2T-O_h_/Si-BC_3_, (c) 2T-S/Si-BC_3_, and (d) 2T-S_h_/Si-BC_3_. The first and second rows show the top and side views of the 4 optimized configurations, respectively; carbon, boron, silicon, oxygen, nitrogen, hydrogen, and sulfur atoms are represented as purple, beige, orange, red, blue, white, and yellow, respectively. The third row displays the calculated band structures and total DOS of each configuration, with the Fermi level set to 0 eV; the high-symmetry k-point circuit runs in the following order: Γ → K → M → Γ. The last row shows the projected DOS of two selected orbitals of each configuration; the Si_3p_, O_2p_, S_3p_, and B_2p_ orbitals are represented as blue, red, green, and yellow, respectively.

**Table 5:** Adsorption Energy *E*_ad_ (eV), Lattice Constant a (Å), Band Gap *E*_g_ (eV), and Binding Distance d (Å) of 2-Thiouracil on Si-BC_3_ ^a^

Complex	*E*_ad_ (eV)	a (Å)	*E*_g_ (eV)	d (Å)
2T-O/Si-BC_3_	–1.854 (che)	10.38	0.294 (ind)	1.810
2T-O_h_/Si-BC_3_	–2.003 (che)	10.29	0.460 (dir)	1.830
2T-S/Si-BC_3_	–0.964 (che)	10.39	0.371 (dir)	3.880
2T-S_h_/Si-BC_3_	–1.213 (che)	10.40	0.374 (dir)	3.490

a Che indicates chemisorption; ind and dir represent indirect and direct band gap, respectively.

#### 3.3.1 2T-O/Si-BC_3_

The optimized atomic structure of 2T-O/Si-BC_3_ is shown in Figure 4a. A chemical bond formed between the O atom and the Si atom, where a bump in the monolayer can be observed at Si. The binding distance is thus only 1.810 Å, as displayed in Table 5, and the C–O–Si bond angle is measured to be 133.4°.

The band structure showcases an indirect band gap of 0.294 eV between the VBM and CBM, as shown in Table 5. This band gap is larger than the 0.124 eV band gap of Si-BC_3_ in Table 3, indicating changes in the electronic structure after 2-thiouracil adsorption. Moreover, the arrow in the band structure points to a flat band at around 0.3 eV, and the total DOS also displays significant electron states at 0.3 eV, demonstrating the influence of the 2-thiouracil molecule on the electronic structure.

The projected DOS in Figure 4a reveals that both the Si_3p_ orbital and the O_2p_ orbital contributed to the states around, below, and above the Fermi level of 0 eV. While evidence of minimal overlapping of orbitals can be seen at all energy levels between −2 and 2 eV, strong hybridization between the Si_3p_ and O_2p_ orbitals can be observed at around 0.3 eV, where the arrow points. This is consistent with the flat band at 0.3 eV and demonstrates evidence of strong adsorption, corresponding to chemisorption and the large adsorption energy of −1.854 eV in Table 5.

#### 3.3.2 2T-O_h_/Si-BC_3_

The optimized atomic structure of 2T-O_h_/Si-BC_3_ is shown in Figure 4b. A bump in the monolayer can be observed at the Si dopant due to the formation of a chemical bond with O, resulting in a short binding distance of 1.830 Å, as shown in Table 5. The C–O–Si bond angle is measured to be 121.5°.

A direct band gap of 0.460 eV, as stated in Table 5, is shown in the band structure between the VBM and CBM at the Γ point. This band gap increased from the 0.124 eV band gap of the Si-BC_3_ monolayer in Table 5, showcasing evidence of adsorption. A flat band indicated by an arrow is displayed at around 0.5 eV, which is confirmed by the significant electron states at 0.5 eV shown in the total DOS, indicating the molecule’s influence on the electronic structure.

The projected DOS in Figure 4b shows the contribution of both the Si_3*p*_ and O_2*p*_ orbitals at, below, and above the Fermi level of 0 eV. Strong hybridization of the two orbitals can be observed above the Fermi level, specifically at 0.5 eV, where the arrow points, corresponding to the flat band at the same energy. This evidence of bonding and chemisorption can be further proven by the large adsorption energy of −2.003 eV shown in Table 5.

#### 3.3.3 2T-S/Si-BC_3_

The optimized atomic structure of 2T-S/Si-BC_3_ is shown in Figure 4c. No chemical bond is observed , and a dip in the monolayer at the Si atom can be seen due to the weak interaction between Si and S and potential repulsion, leading to a large binding distance of 3.880 Å, as shown in Table 5. The repulsion may be due to the low reactivity and large atomic size of the S atom.

From the band structure, a direct band gap between the VBM and CBM at the Γ point can be observed. After 2-thiouracil adsorption, the band gap of Si-BC_3_ increased from 0.124 to 0.372 eV, as shown in Tables 3 and 5. This demonstrates some influence of 2-thiouracil on the electronic structure of the complex.

The projected DOS in Figure 4c shows that the S_3p_ orbital only contributed to electron states below the Fermi level, while the Si_3p_ orbital contributed at, below, and above the Fermi level of 0 eV. Moreover, there is very minimal hybridization between the two orbitals below the Fermi level, between −1 eV and −0.5 eV, demonstrating weak adsorption and corresponding to the smaller adsorption energy of −0.964 eV displayed in Table 5. Although less interaction is observed compared to that of other configurations, 2T-S/Si-BC_3_ still demonstrates chemisorption.

#### 3.3.4 2T-S_h_/Si-BC_3_

The atomic structure of 2T-S_h_/Si-BC_3_ is shown in Figure 4d. In this configuration, the molecule is horizontal, and the S atom was originally placed closest to the Si atom; however, the S atom moved away during relaxation onto a B atom, possibly due to the repulsion between S and Si. Moreover, the monolayer also displayed a dip at the Si atom, potentially to relieve strain and reach a lower energy state. Therefore, as shown in Table 5, the binding distance between the molecule and the monolayer is 3.490 Å.

A direct band gap between the VBM and CBM at the Γ point is shown in the band structure. After 2-thiouracil adsorption, the band gap of Si-BC_3_ increased from 0.124 to 0.374 eV, as shown in Tables 3 and 5, indicating some influence of the 2-thiouracil molecule.

In Figure 4d, the projected DOS reveals that the S_3p_ orbital contributes only to electron states below the Fermi level, while the B_2p_ orbital contributes to states at, below, and above the Fermi level of 0 eV. Furthermore, S_3p_ and B_2p_ orbitals displayed some overlap between −2 eV and −1 eV, showcasing some adsorption. The adsorption energy of −1.213 eV in Table 5 further classifies this adsorption as chemisorption.

All molecule-monolayer complexes demonstrate chemisorption. Specifically, we observed that 2T-O/Si-BC_3_ and 2T-O_h_/Si-BC_3_ resulted in the formation of chemical bonds, short binding distances, the presence of flat bands, and the largest adsorption energies; projected DOS analysis also showcases strong hybridization between the 2-thiouracil molecule and the Si-BC_3_ monolayer. Among the two, 2T-O_h_/Si-BC_3_ demonstrated the largest adsorption energy of −2.003 eV. We also observed that in configurations where S was selected as the interacting atom with the monolayer, 2T-S/Si-BC_3_ and 2T-S_h_/Si-BC_3_, the Si dopant dipped below the monolayer, resulting in long binding distances, small adsorption energies, and minimal hybridization between the molecule and monolayer. Therefore, the O atom on 2-thiouracil has stronger chemisorption to the Si dopant on Si-BC_3_ than the S atom.

### 3.4 2-Thiouracil Adsorbed on Al-Doped BC_3_

Because placing molecules onto the Al-dopant has shown the strongest adsorption in previous studies,^36,37^ the 2-thiouracil was placed onto Al-doped BC_3_ in three positions: 2T-O/Al-BC_3_, 2T-O_*h*_/Al-BC_3_, and 2T-S_h_/Al-BC_3_. O and S represent the atom on the molecule interacting with the Al dopant on the monolayer; h indicates a horizontal molecule configuration as opposed to vertical. Top and side views of their optimized atomic structures, band structures, and total and projected DOS are shown in Figure 5. Adsorption energy, lattice constant, band gap, and binding distance of each complex are shown in Table 6.

**Figure 5:**
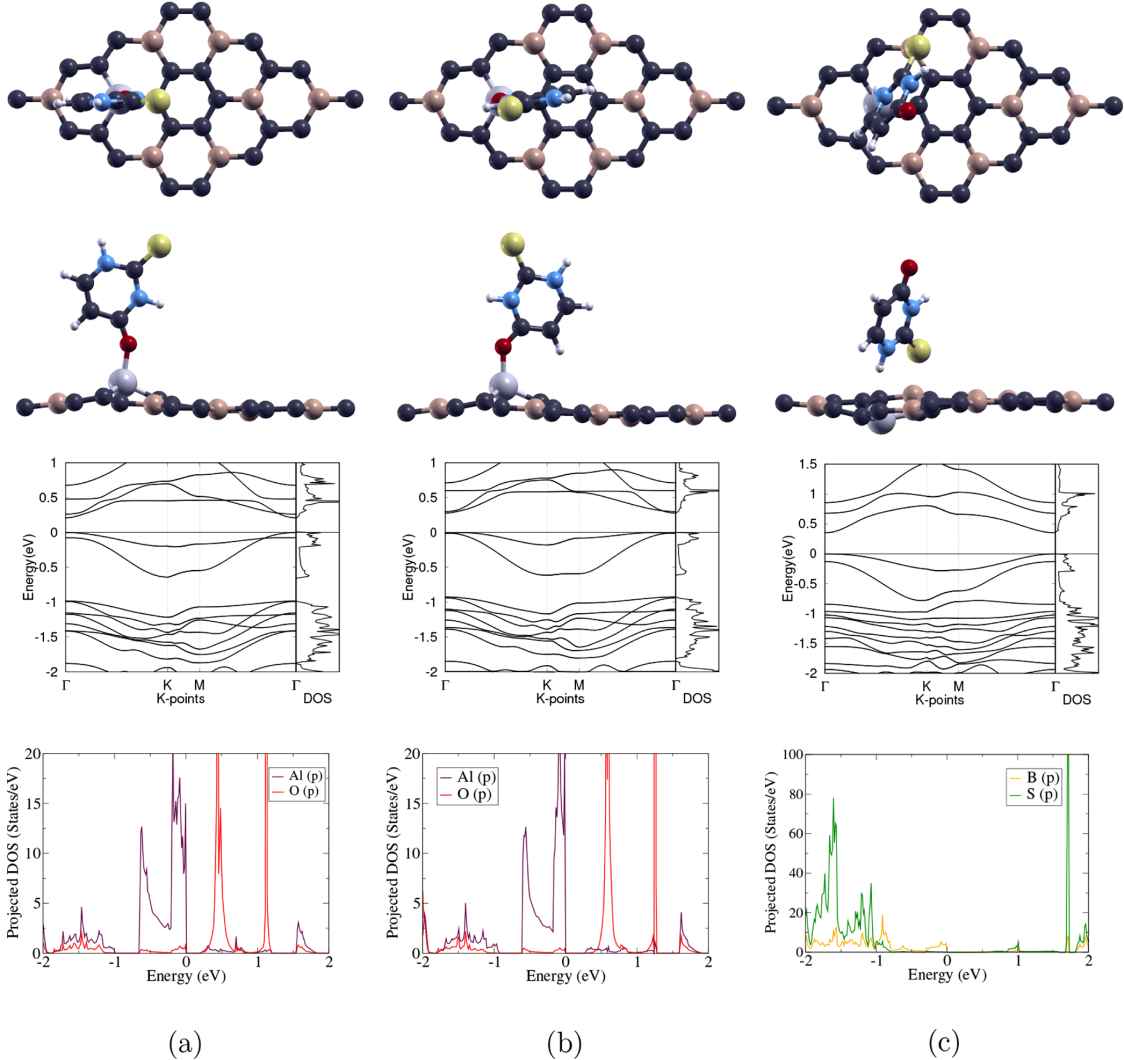
Optimized configurations of the 2-thiouracil molecule in 3 different positions on the Al-doped BC_3_ monolayer: (a) 2T-O/Al-BC_3_, (b) 2T-O_h_/Al-BC_3_, and (c) 2T-S_h_/Al-BC_3_. The first and second rows show the top and side views of the 3 optimized configurations, respectively; carbon, boron, aluminum, oxygen, nitrogen, hydrogen, and sulfur atoms are represented as purple, beige, gray, red, blue, white, and yellow, respectively. The third row displays the calculated band structures and total DOS of each configuration, with the Fermi level set to 0 eV; the high-symmetry k-point circuit runs in the following order: Γ → K → M → Γ. The last row shows the projected DOS of two selected orbitals of each configuration; the Al_3p_, O_2p_, S_3p_, and B_2p_ orbitals are represented as maroon, red, green, and yellow, respectively.

**Table 6:** Adsorption Energy *E*_ad_ (eV), Lattice Constant a (Å), Band Gap *E*_g_ (eV), and Binding Distance d (Å) of 2-Thiouracil on Al-BC_3_ ^a^

Complex	*E*_ad_ (eV)	a (Å)	*E*_g_ (eV)	d (Å)
2T-O/Al-BC_3_	–1.669 (che)	10.51	0.212 (dir)	1.880
2T-O_h_/Al-BC_3_	–1.898 (che)	10.46	0.282 (dir)	1.890
2T-S_h_/Al-BC_3_	–1.210 (che)	10.46	0.353 (dir)	2.130

a Che indicates chemisorption , and dir represents the direct band gap.

#### 3.4.1 2T-O/Al-BC_3_ and 2T-O_*h*_/Al-BC_3_

The optimized structure of 2T-O/Al-BC_3_ is shown in Figure 5a. In 2T-O_h_/Al-BC_3_, shown in Figure 5b, the molecule was initially placed horizontally but became vertical after relaxation. Notably, the configuration is very similar to that of 2T-O/Al-BC_3_, demonstrating that a vertical configuration of 2-thiouracil is energetically favorable. Both configurations display the formation of a chemical bond between O and Al, resulting in a bump in the monolayer at Al. For 2T-O/Al-BC_3_ and 2T-O_*h*_/Al-BC_3_, the binding distances are 1.880 and 1.890 Å, respectively, as shown in Table 6. The C–O–Al bond angles are 151.4° and 137.4° for 2T-O/Al-BC_3_ and 2T-O_h_/Al-BC_3_, respectively.

In both band structures, direct band gaps between the VBM and CBM at the Γ point are shown. Both the band gaps of 2T-O/Al-BC_3_ and 2T-O_h_/Al-BC_3_, 0.212 and 0.282 eV, respectively, are larger than the 0.166 eV band gap of Al-BC_3_, as shown in Tables 6 and 3. This showcases changes in the electronic structures of the two configurations after 2-thiouracil adsorption. Both band structures also showcase a partially flat band at around 0.5 eV, corresponding to significant electron states in the total DOS at the same energy level. This indicates the influence of the molecule on the electronic structures of both complexes.

In the projected DOS for both 2T-O/Al-BC_3_ in Figure 5a and 2T-O_h_/Al-BC_3_ in Figure 5b, the Al_3p_ orbital contributed mostly to the states around the Fermi level, but both the Al_3p_ and the O_2p_ orbitals contributed to states above and below the Fermi level. Moreover, hybridization can be observed between −2 and −1 eV as well as around 1.6 eV between the Al_3p_ and O_2p_ orbitals, demonstrating evidence of adsorption and bonding. This projected DOS analysis corresponds to chemisorption and large adsorption energies of −1.669 eV (2T-O/Al-BC_3_) and −1.898 eV (2T-O_h_/Al-BC_3_).

#### 3.4.2 2T-S_h_/Al-BC_3_

The optimized structure of 2T-S_h_/Al-BC_3_ is shown in Figure 5c. After adsorption, the sulfur atom moved away from the Al atom onto the B atom, potentially due to the high-energy interaction and repulsion between S and Al. Furthermore, 2T-Al-BC_3_–S in Figure 5c showed no new bond and displayed a dip in the monolayer at the Al atom, providing further evidence of repulsion for energy favorability. In Table 6, the binding distance between the 2-thiouracil molecule and the Al-BC_3_ monolayer is 2.130 Å, which is larger than the other two configurations. Notably, another structure where S was interacting with Al on the monolayer was originally computed and resulted in the departure of the 2-thiouracil molecule from the monolayer, further demonstrating that the S atom would repel the Al dopant on the monolayer.

The direct band gap of 2T-S_h_/Al-BC_3_ is between the VBM and CBM at the Γ point. After 2-thiouracil adsorption, the band gap of Al-BC_3_ increased from 0.166 to 0.353 eV, as shown in Tables 3 and 6, demonstrating a change in electronic structure after adsorption.

The projected DOS in Figure 5c shows the contributions of both B_2p_ and S_3p_ orbitals to the electron states below and above the Fermi level, but at the Fermi level, only B_2p_ contributes to the states. Moreover, some hybridization can be observed between −2 eV and −1 eV, demonstrating interactions corresponding to the adsorption energy of −1.210 eV in Table 6. Adsorption energy and hybridization indicate evidence of chemisorption.

Overall, all configurations demonstrate chemisorption. 2T-O/Al-BC_3_ and 2T-O_h_/Al-BC_3_ resulted in very similar configurations, and both complexes displayed the formation of bonds, short binding distances, the presence of flat bands, and large adsorption energies. Projected DOS analysis also showcases evidence of hybridization between the orbitals of the molecule and the monolayer, demonstrating strong adsorption when O is placed on top of Al. We observed that when the S atom interacts with the Al atom on the monolayer in 2T-S_h_/Al-BC_3_, the Al atom dips below the monolayer, resulting in small adsorption energies and long binding distances. The lack of hybridization also indicates weaker chemisorption. Therefore, similar to the configurations of the Si-BC_3_ monolayer, it was observed that the O atom on 2-thiouracil has stronger chemisorption on Al than the S atom.

### 3.5 Charge Transfer

As 2T-O/Si-BC_3_, 2T-O_h_/Si-BC_3_, 2T-O/Al-BC_3_, and 2T-O_h_/Al-BC_3_ demonstrated successful adsorption, we wanted to conduct charge transfer calculations using eq 3 to further investigate the electron density distribution caused by the adsorption of 2-thiouracil.

The charge difference between the molecule and monolayers was calculated, and the isosurfaces for each configuration were plotted to visually represent the charge transfer results. The isosurfaces of 2T-O/Si-BC_3_, 2T-O_*h*_/Si-BC_3_, 2T-O/Al-BC_3_, and 2T-O_*h*_/Al-BC_3_ are displayed in Figure 6a,b,c and d, respectively. Under the same isovalue of 0.004 e/Bohr^3^, the 2T/Si-BC_3_ configurations visibly had more charge transfer than the 2T-Al-BC_3_ configurations. This further demonstrates that the Si–O bond is stronger than the Al–O bond.

**Figure 6:**
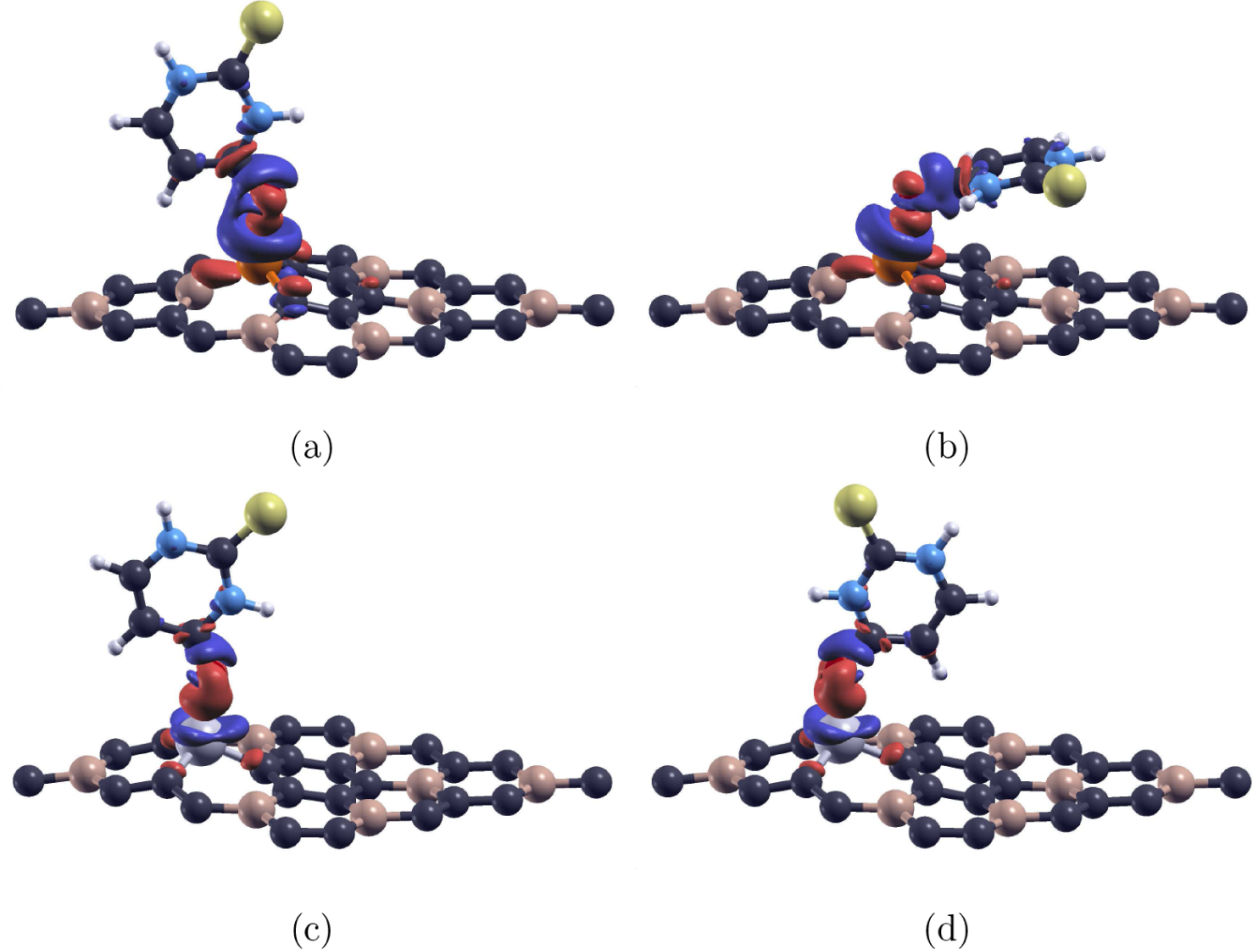
Charge transfer of the 2-thiouracil molecule adsorbed on Si-doped and Al-doped BC_3_ monolayers. (a) 2T-O/Si-BC_3_, (b) 2T-O_*h*_/Si-BC_3_, (c) 2T-O/Al-BC_3_, and (d) 2T-O_h_/Al-BC_3_. The isovalue across all charge density plots = 0.004 e/Bohr^3^. Red regions indicate charge accumulation, and blue regions indicate charge depletion. Carbon, boron, silicon, aluminum, oxygen, nitrogen, hydrogen, and sulfur atoms are represented as purple, beige, orange, gray, red, blue, white, and yellow, respectively.

In Figure 6, all four configurations displayed considerable charge transfer. Red regions represent charge accumulation, and blue regions represent charge depletion. We observed that the 2-thiouracil molecule, especially the O atom, always gains electron charge, while the doped BC_3_ monolayers lose charge at the dopant. This may be due to the high electronegativity and, thus, reactivity of the O atoms compared to the dopants. Therefore, the considerable charge transfer between the molecule and monolayer confirms the O atom’s strong chemisorption to the doped BC_3_ monolayers.

## 4 Conclusion

Using first-principles calculations based on DFT, this study aimed to discover a novel drug delivery carrier for the antithyroid drug 2-thiouracil using pristine and doped graphene-like BC_3_ monolayers. Our results indicate that the adsorption strength of 2-thiouracil follows the trend: Si-doped BC_3_ > Al-doped BC_3_ > pristine BC_3_. 2-Thiouracil on pristine BC_3_ displays physisorption in all configurations, while the molecule-monolayer complexes of both doped monolayers demonstrate chemisorption. When placing the 2-thiouracil molecule on doped BC_3_ monolayers, the O atom demonstrated stronger adsorption to the dopants than the S atom, primarily due to the O atom’s high reactivity. Moreover, Si-doped BC_3_ demonstrated more stable configurations and stronger adsorption of the 2-thiouracil molecule than Al-doped BC_3_. The stronger interaction between 2-thiouracil and the Si-doped BC_3_ monolayer enhances the stability of the complex and reduces the risk of premature release during circulation. Environmental conditions at the thyroid site can weaken these interactions, allowing the controlled release of 2-thiouracil. Among all configurations, 2T-O_h_/Si-BC_3_ showed the largest adsorption energy of −2.003 eV, making Si the most effective dopant and the horizontal O-binding configuration the most promising for targeted drug delivery. Although these results are based on first-principles calculations, they suggest that substituent doping of BC_3_ with Si could create a stable binding site for the O atom on 2-thiouracil. Future work should evaluate biocompatibility, pH dependence, and stability in aqueous environments of the complex to assess its potential as a drug delivery platform for thyroid treatment.
